# Supplementary material for: Mitochondrial MsrB2 serves as a switch and transducer for mitophagy
Source: EMBO Mol Med. 2019 Jul 8;11(8):e10409. doi: 10.15252/emmm.201910409 (PMC6685081; doi:10.15252/emmm.201910409)

Appendix Table of Contents

1. Appendix Table S1-S2

2. Appendix figure legends S1-S5

3. Appendix figures S1-S5

Appendix Tables

Appendix Table S1. Clinical characteristics of healthy control and patients with type 2 DM

	Healthy control	T2DM
	n=8	n=66
Age (years ± SD)	41.5±6.3	57.5±12.6
Gender (Males/Females)	8/0	40/26
BMI (kg/m^2 ± SD)	21.8±2.0	34.1±8.6
Blood glucose (mg/dL ± SD)	103.8±4.7	170.7±66.8
HbA1c (% ± SD)	5.2±0.3	7.2±1.8
Systolic blood pressure (mmHg ± SD)	114.1±5.8	134.6±14.7
Diastolic blood pressure (mmHg ± SD)	77.4±3.5	74.7±12.7
Hypertension (%)	0/8 (0%)	47/66 (71.2%)
CAD (%)	0/8 (0%)	17/66 (25.8%)
Medications (%)		
Aspirin	0/8 (0%)	32/66 (48.5%)
Clopidogrel	0/8 (0%)	3/66 (4.5%)
Warfarin	0/8 (0%)	4/66 (6.1%)
Statin	0/8 (0%)	41/66 (62.1%)
Beta-blocker	0/8 (0%)	30/66 (45.5%)
Angiotensin converting enzyme inhibitor	0/8 (0%)	26/66 (39.4%)
Angiotensin receptor blocker	0/8 (0%)	14/66 (21.2%)
Insulin	0/8 (0%)	30/66 (45.5%)
Metformin	0/8 (0%)	21/66 (31.8%)
Other anti-diabetic drugs	0/8 (0%)	8/66 (12.1%)
Diuretic	0/8 (0%)	21/66 (31.8%)

Appendix Table S2. Antibody information

Company	Target	Cat#	Lot#	Clone #	Species	Titer
Cell Signaling	Cleaved Caspase3	9661s	43	D175	Rabbit	1:1000
	Cytochrome C	4272S	6		Rabbit	1:1000
	GAPDH	2118L	8		Rabbit	1:1000
	Parkin	4211S	4		Mouse	1:1000
	pp53(S15)	9284			Rabbit	1:1000 (WB)<br>1:500 (IF)
	P53	2524			Mouse	1:1000
	Ubiqitin	3936S	9		Mouse	1:1000
	MsrB2	Home made		KO mice tissue tested	Rabbit	1:1000
Abcam	LC3	Ab48394			Rabbit	1:500
	Parkin	Ab15954			Rabbit	1:500
	MsrB2	Ab101513			Rabbit	1:500
	MsrB1	Ab71175			Rabbit	1:100
	MsrB3	Ab88731			Rabbit	1:100
	MsrA	16803			Rabbit	1:1000
Cosmo	LC3			LC3 1703	Mouse	1:100 (IF)
Santa cruze	Cox4	SC-69360		G-20	goat	1:100 (IF)
Sigma	B-actin	A5316		AC-74	mouse	1:5000

Appendix figure legends

Appendix fig S1. Identification of MsrB2 as LC3 interaction protein in human platelets.

A. Immunoprecipitation (IP) of LC3 in human platelet w/ and w/o CCCP treatment. Western blot analysis of LC3 and silver staining for LC/mass analysis.

B. LC3 interacting motif (LIF) in human and mouse MsrB2 protein sequence.

C. Western blot analysis of Methionine sulfoxide reductase (Msr) A, B1, B2 and B3 in human Healthy Control (HC; 4 HC pooled) and Diabetic mellitus (DM; 10 DM pooled) platelets.

Appendix fig S2. Expression profiles of apoptosis proteins in MsrB2 KO mice platelets.

A. Isolated mice washed platelets used for apoptosis array. Platelets from 5 WT mice and 8 MsrB2 KO mice (CRISPR-Cas9 MsrB2 KO) in each group were pooled. Expression profiles in WT and MsrB2 KO platelets using mouse apoptosis array (R&D system). The intensity of the dot blots were analyzed by Image J software. Relative expression of apoptosis proteins in WT and MsrB2 KO platelets were displayed by fold change (compare dot intensity of MsrB2 KO with WT KO).

B. Western blot analysis of Methionine sulfide reductase B2 (MsrB2) and Cytochrom C in WT and MsrB2 KO mice platelets. The nonparametric *t* test was performed for comparisons of 2 groups. Analysis was performed with Prism software (GraphPad Software, Inc, La Jolla, CA). A difference of $P<0.05$ was considered significant. (CytoC; ***p=0.0051* vs. WT, n-=4).

Appendix fig S3. LC3 interacting peptide (LIF) increase HG induced apoptosis in MEG-01 cells through reduction of MsrB2 function.

A. Amino acid sequence of control (Tat-pep CP) and MsrB2 LIF motif peptides (LP) used for treatment.

B. Western blot analysis of MsrB2, pp53(s15) and active caspasee3 in MEG-01 cells after cell penetration peptide treatment.

Appendix fig S4. MsrB2 and mitophagy increased by oxidative stress in human plateles.

A. Western blot analysis of LC3I/II, MsrB2 and Parkin in HC platelets treated with H_2O_2 (1 mM for 1 hr) alone or with NAC (100 μM for 30 min). GAPDH was used as the loading control.

B. Quantification analysis of LC3I/II, MsrB2 and Parkin (LC3II; $^*p=0.0382$, MsrB2; $^*p=0.0269$ and Parkin; $^*p=0.0208$ vs. HC group in H_2O_2, LC3II; $^*p=0.0375$, MsrB2; $^*p=0.0156$ and Parkin; $^*p=0.0421$ in H_2O_2/NAC vs. H_2O_2 group, n=3 for each group).

C. Western blot analysis of polyubiquitination in HC platelets treated with H_2O_2 (1 mM for 1 hr) alone or with NAC (100 μM for 30 min).

D. Western blot analysis of polyubiquitination in in HC (n=3) and DM (n=9) platelets**.** Quantification analysis on HC (n=3) and DM (n=9) individuals. ($^*p=0.0122$ vs. HC). Actin (same sample with fig 5C) served as the loading control.

E. MsrB2 ubiquitination assay after transient transfection of MsrB2-GFP (1μg) w/ or w/o CCCP in HEK293. After transfection (48hrs), IP was performed using GFP-Trap bead, followed by Western blot analysis using UB and MsrB2 antibody. The nonparametric *t* test was performed for comparisons of 2 groups. Analysis was performed with Prism

software (GraphPad Software, Inc, La Jolla, CA). A difference of $P<0.05$ was considered significant.

Appendix fig S5. MsrB2 released out from Mitochondria

A. Confocal microscopy was used for co-staining after transient transfection using MsrB2-GFP and CFP-Mito plasmid DNA into H9C2 cell. After 48hrs incubation and then treated 10 μM CCCP or DMSO. Small boxes indicate enlarged sites of colocalization of Parkin, LC3 and MsrB2 in CCCP-1 and CCCP-b panel. Arrow indicates released MsrB2 in mitochondria.

B. Graph indicates colocalization between MsrB2 and Mito signal. Colocalization efficiency verified in Velocity program of each cell in each group was converted to fold and compared with HC values (**$p<0.0001$ vs. HC, n=3). The nonparametric t test was performed for comparisons of 2 groups. Analysis was performed with Prism software (GraphPad Software, Inc, La Jolla, CA). A difference of $P<0.05$ was considered significant.

Appendix fig. S1.

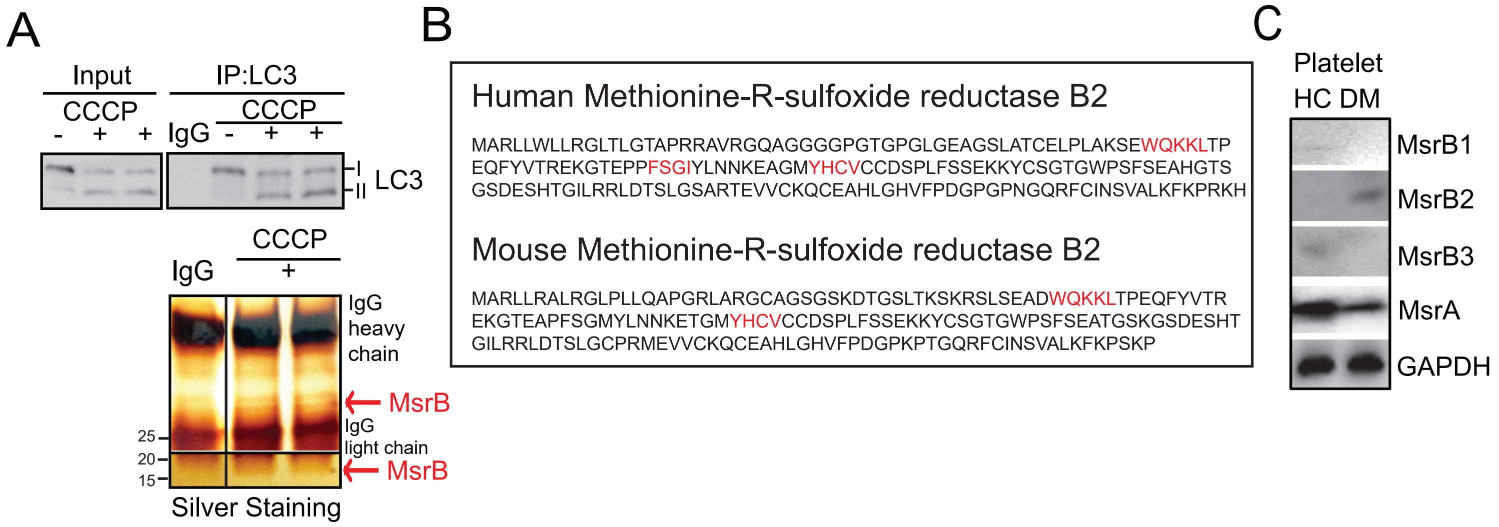

Appendix fig. S2

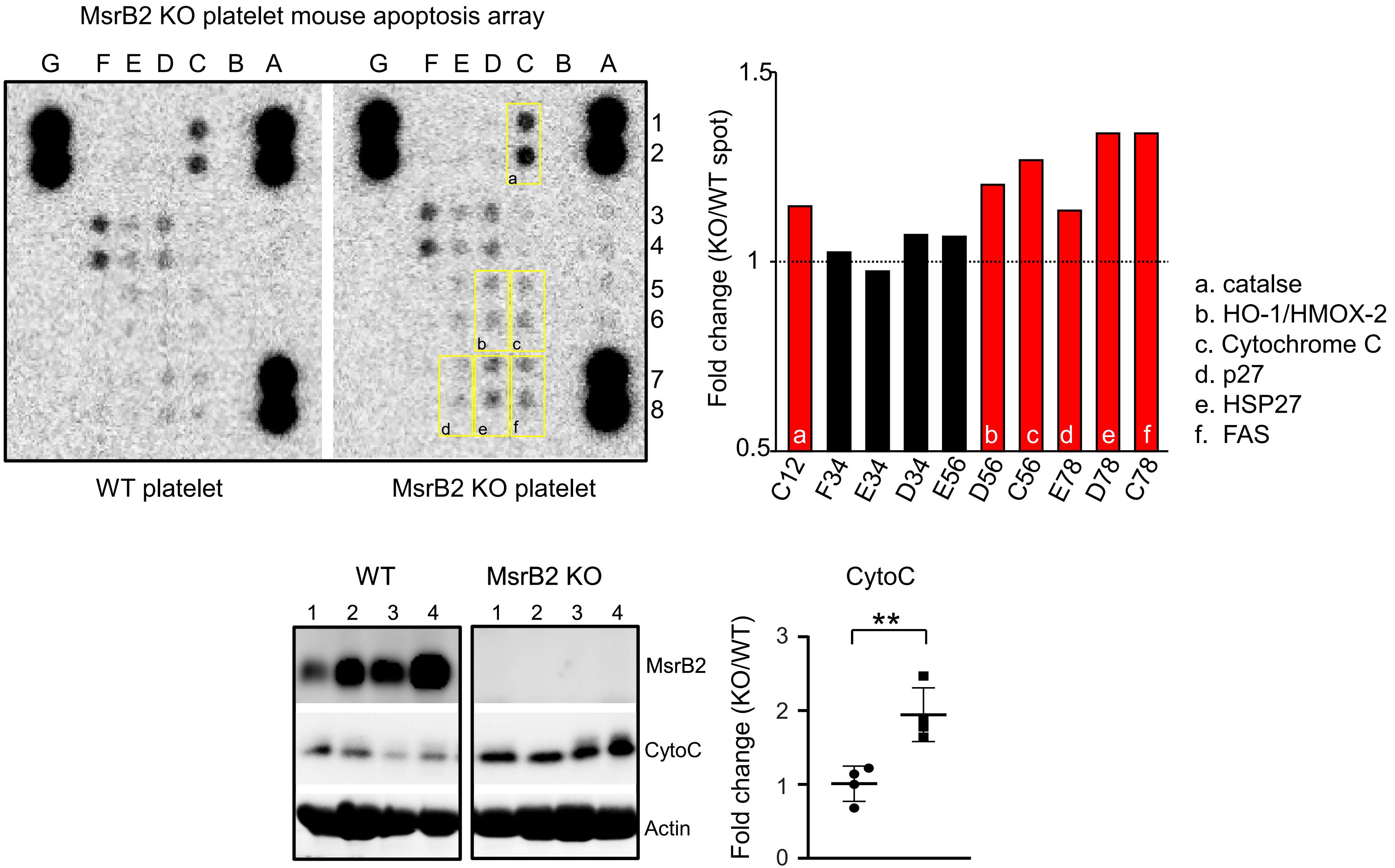

Appendix fig. S3

A

Tat-pep (CP) : NH_2-GRKKRRQRRRPPQ-COOH

MsrB2 LIF-pep (LP) : NH_2-GRKKRRQRRRPPQ-KSE WQKKL TPE-COOH

+ NH_2-GRKKRRQRRRPPQ-AGM YHCV CCD-COOH

B

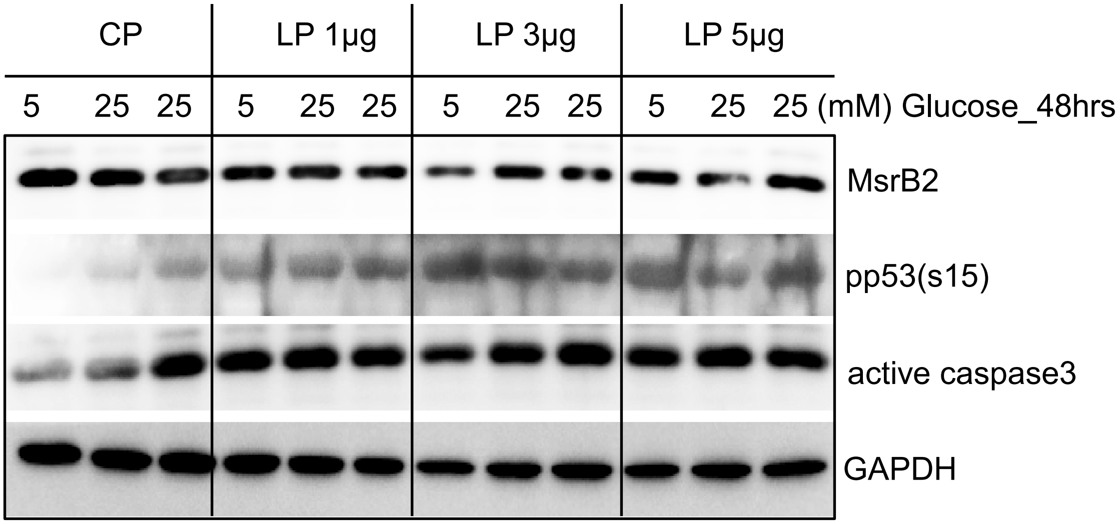

Appendix fig. S4

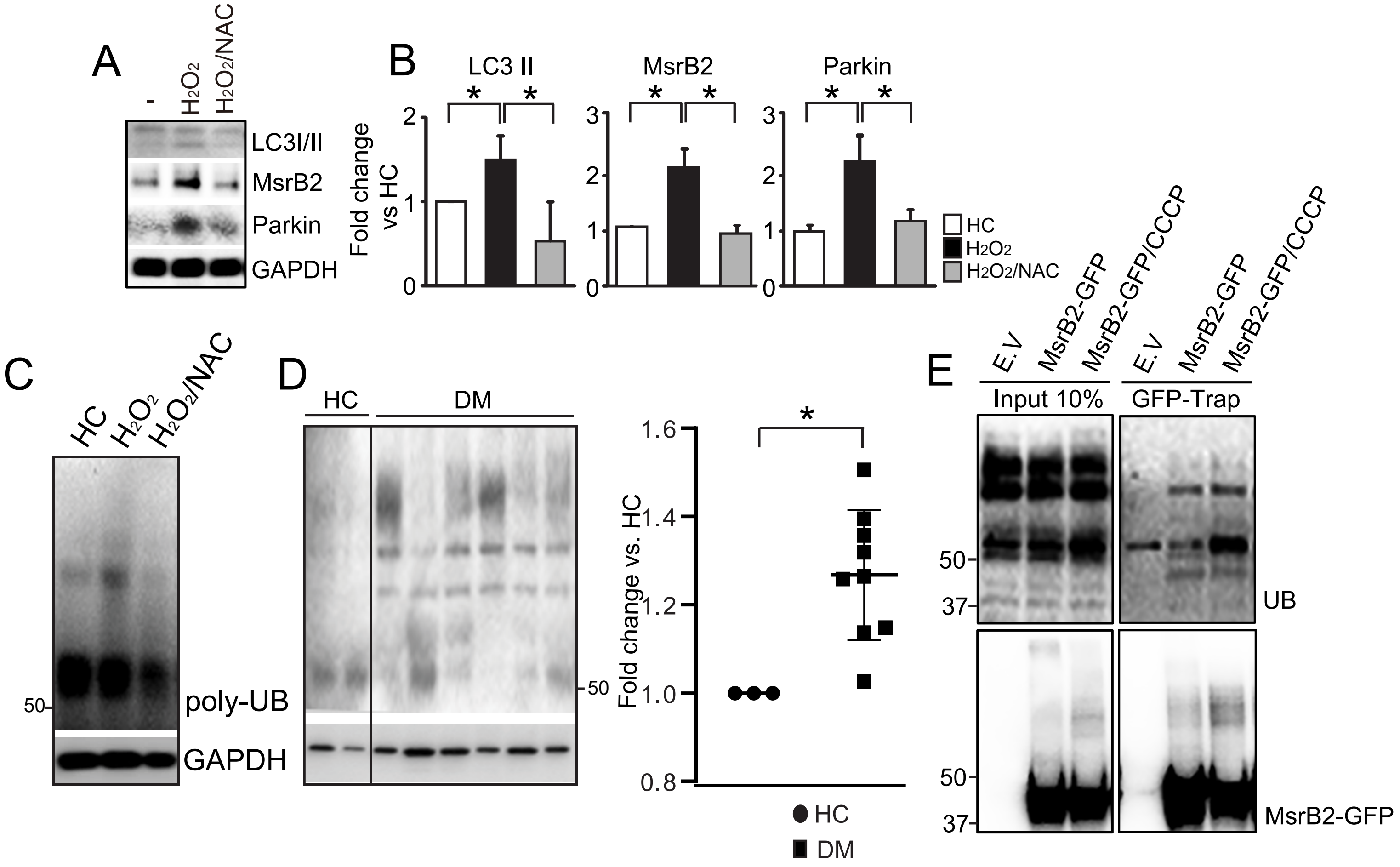

Appendix fig. S5

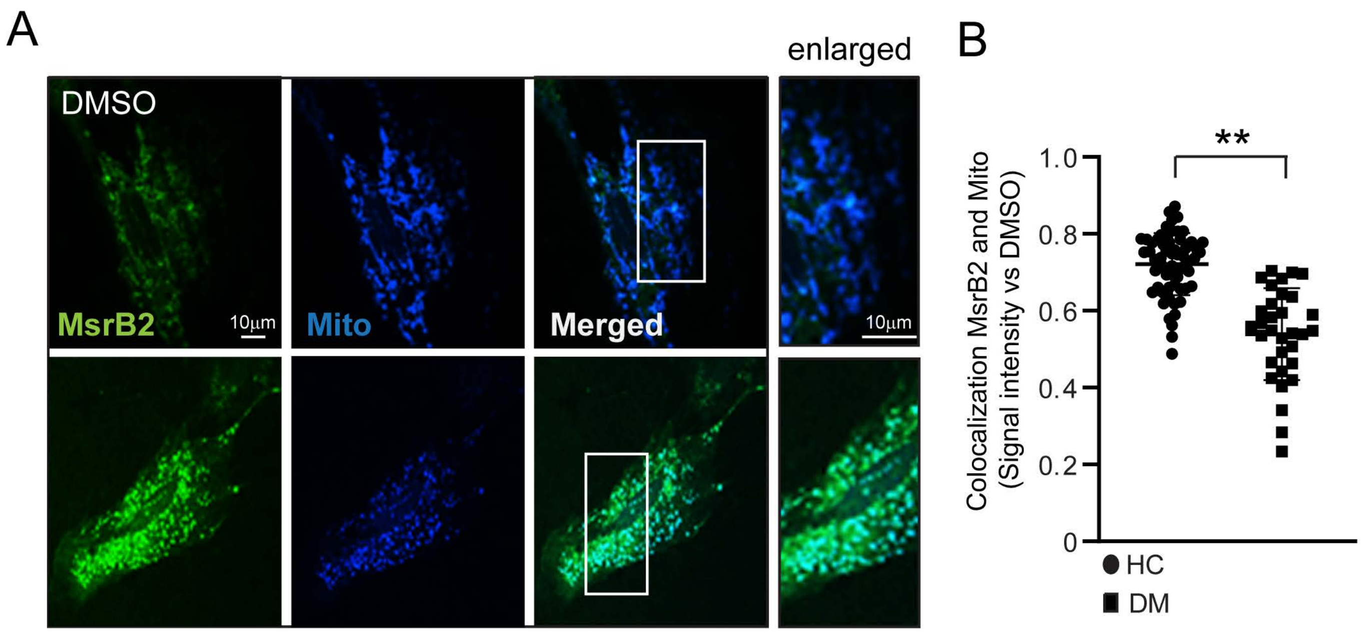

Supplement: Supplementary file 1 — Appendix [file EMMM-11-e10409-s001.pdf]
