# Supplementary material for: Mitochondrial MsrB2 serves as a switch and transducer for mitophagy
Source: EMBO Mol Med. 2019 Jul 8;11(8):e10409. doi: 10.15252/emmm.201910409 (PMC6685081; doi:10.15252/emmm.201910409)

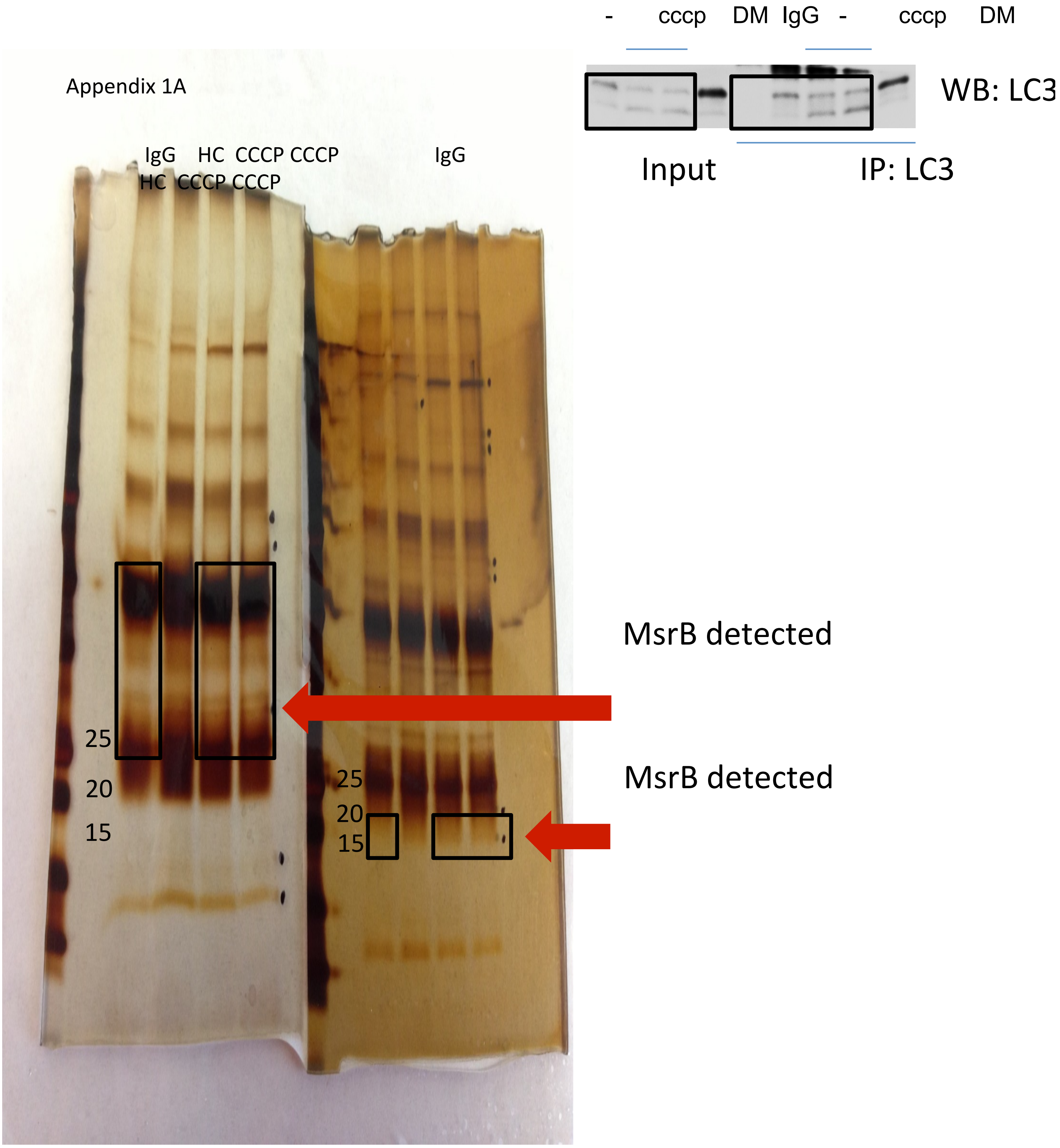
- cccp DM IgG - cccp DM
WB: LC3
Input
IP: LC3
Appendix 1A
IgG HC CCCP CCCP
HC CCCP CCCP
IgG
25
20
15
25
20
15
MsrB detected
MsrB detected

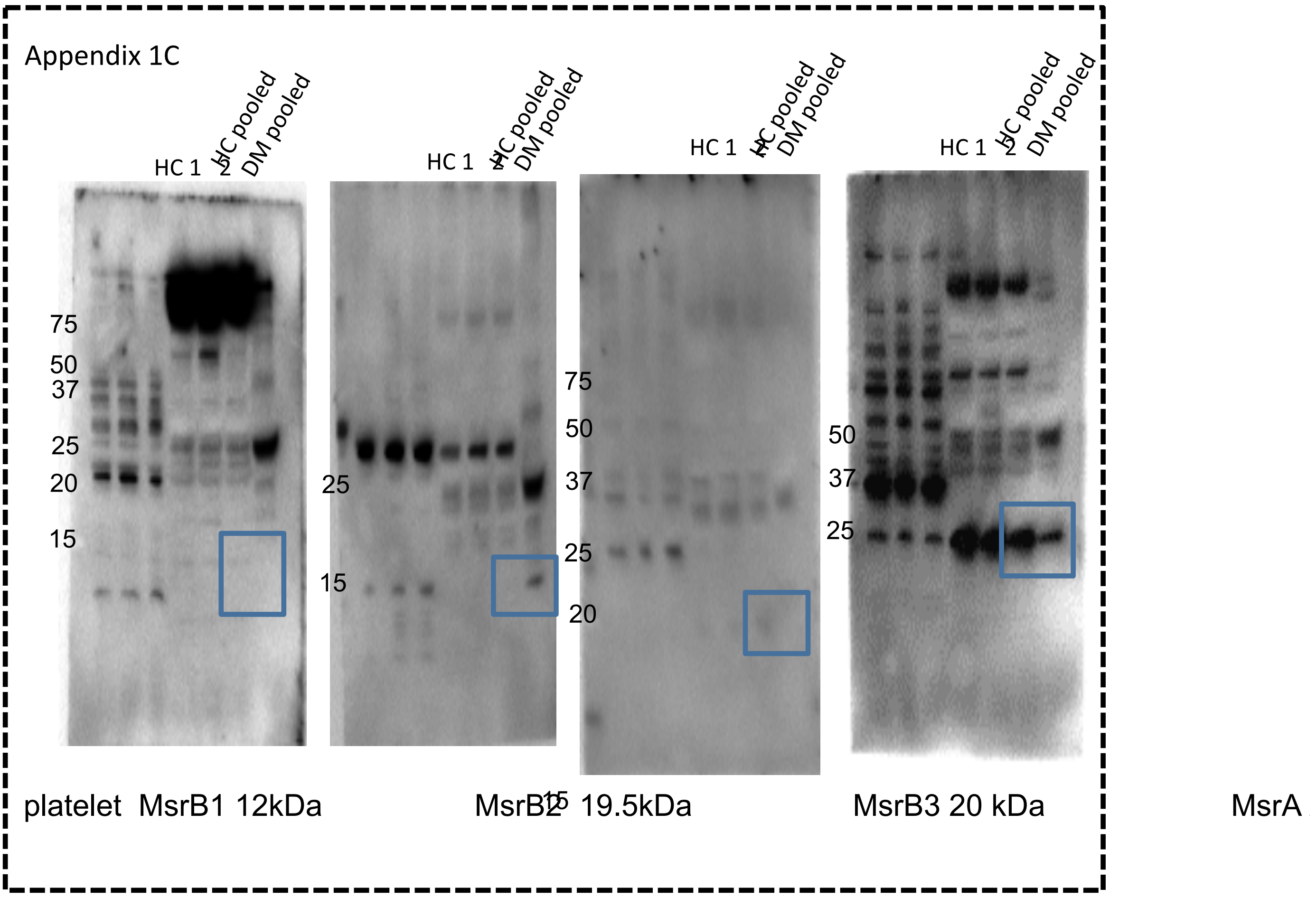
Appendix 1C
HC 1
2
HC pooled
DM pooled
75
50
37
25
20
15
platelet MsrB1 12kDa
MsrB2 19.5kDa
MsrB3 20 kDa
MsrA

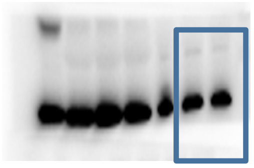

GAPDH

Appendix 4D

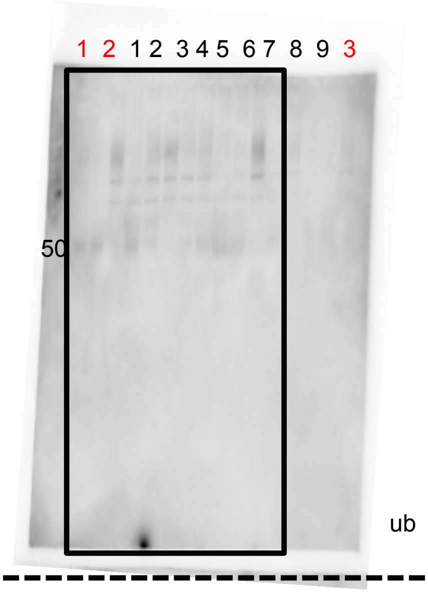

HC =3

DM = 9

Appendix 5

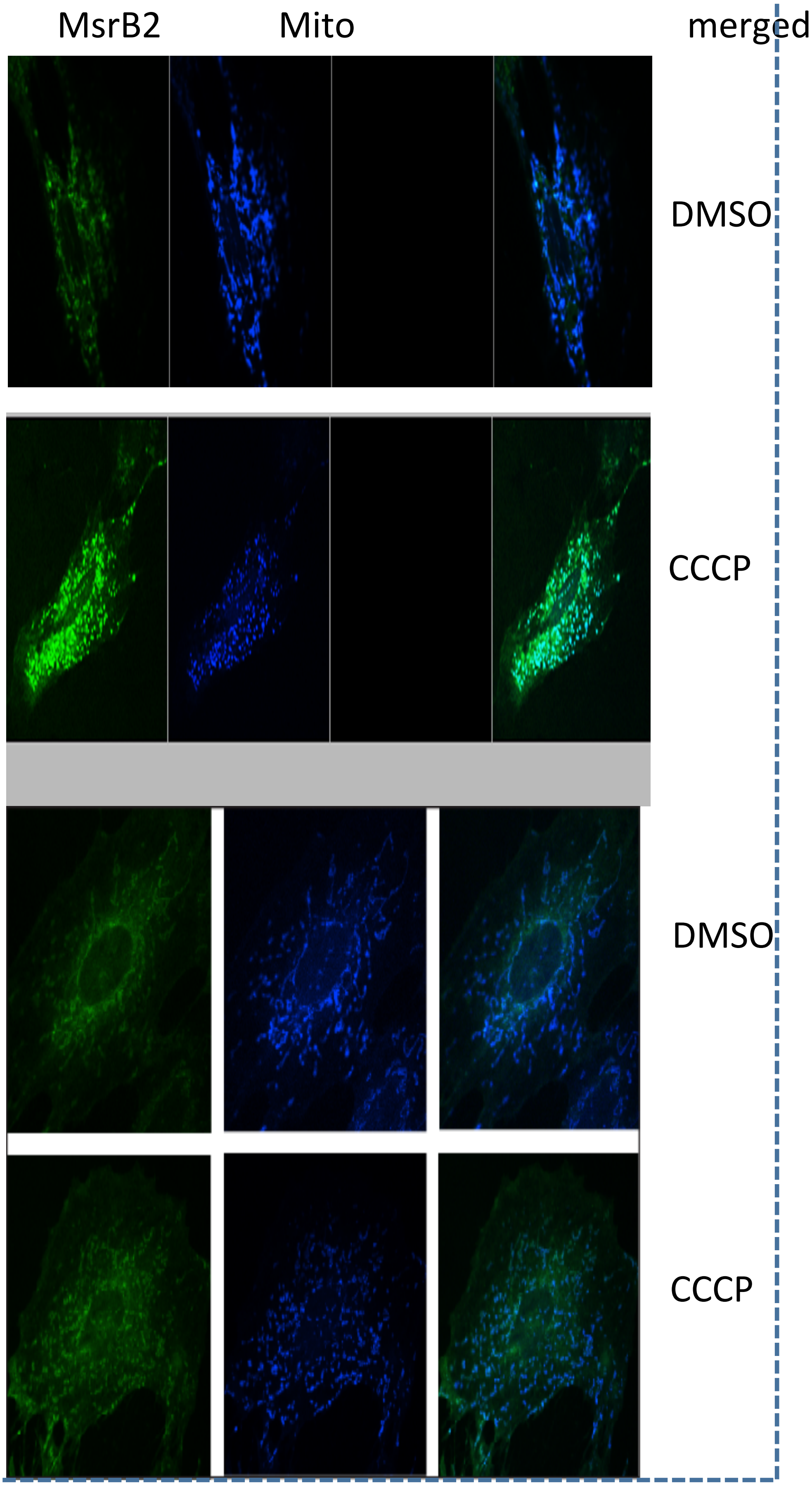

Supplement: Supplementary file 2 — Source Data for Appendix [file EMMM-11-e10409-s004.zip › emmm201910409-sup-0004-SDataAppendixFigs/emmm201910409-sup-0004-SDataAppendixFigs.pdf]
