# Supplementary figures and images for: Mitochondrial MsrB2 serves as a switch and transducer for mitophagy
Source: EMBO Mol Med. 2019 Jul 8;11(8):e10409. doi: 10.15252/emmm.201910409 (PMC6685081; doi:10.15252/emmm.201910409)

Figure2A

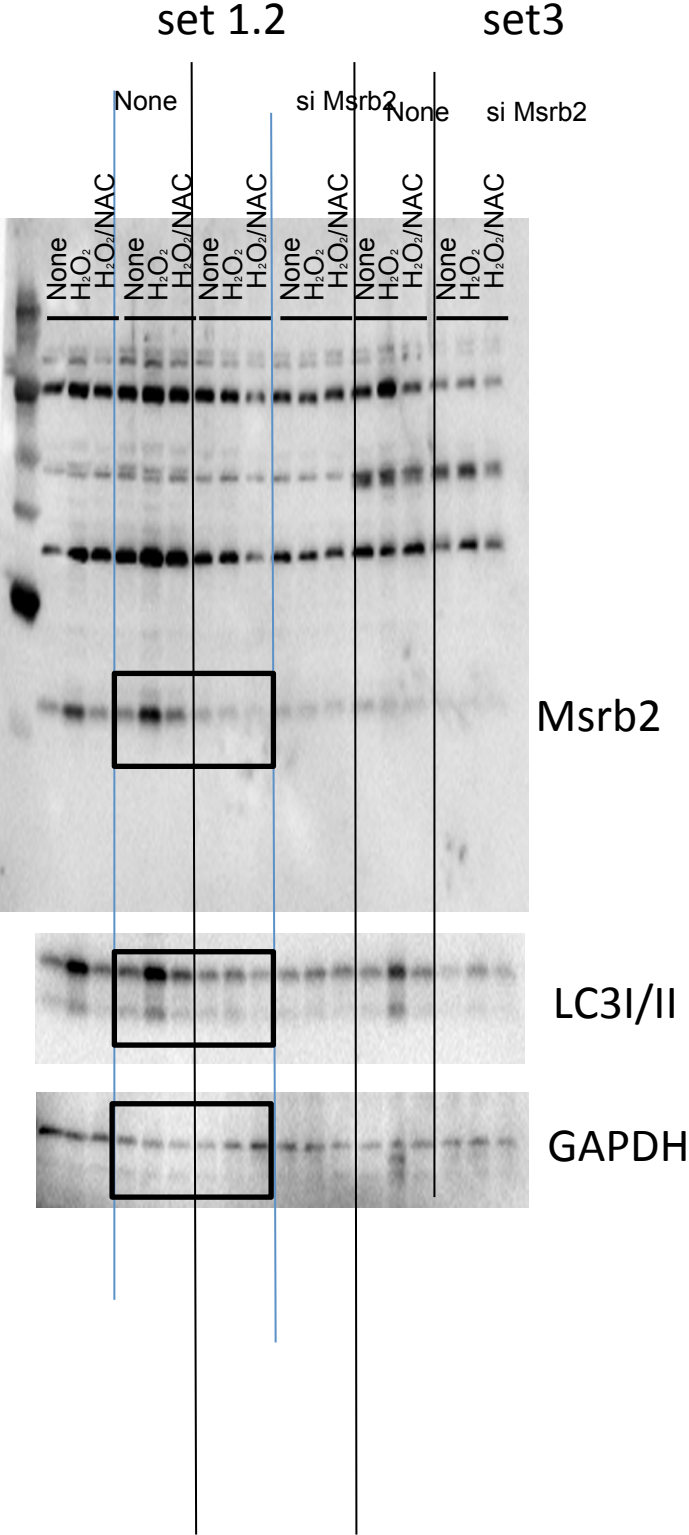

**Figure2B**

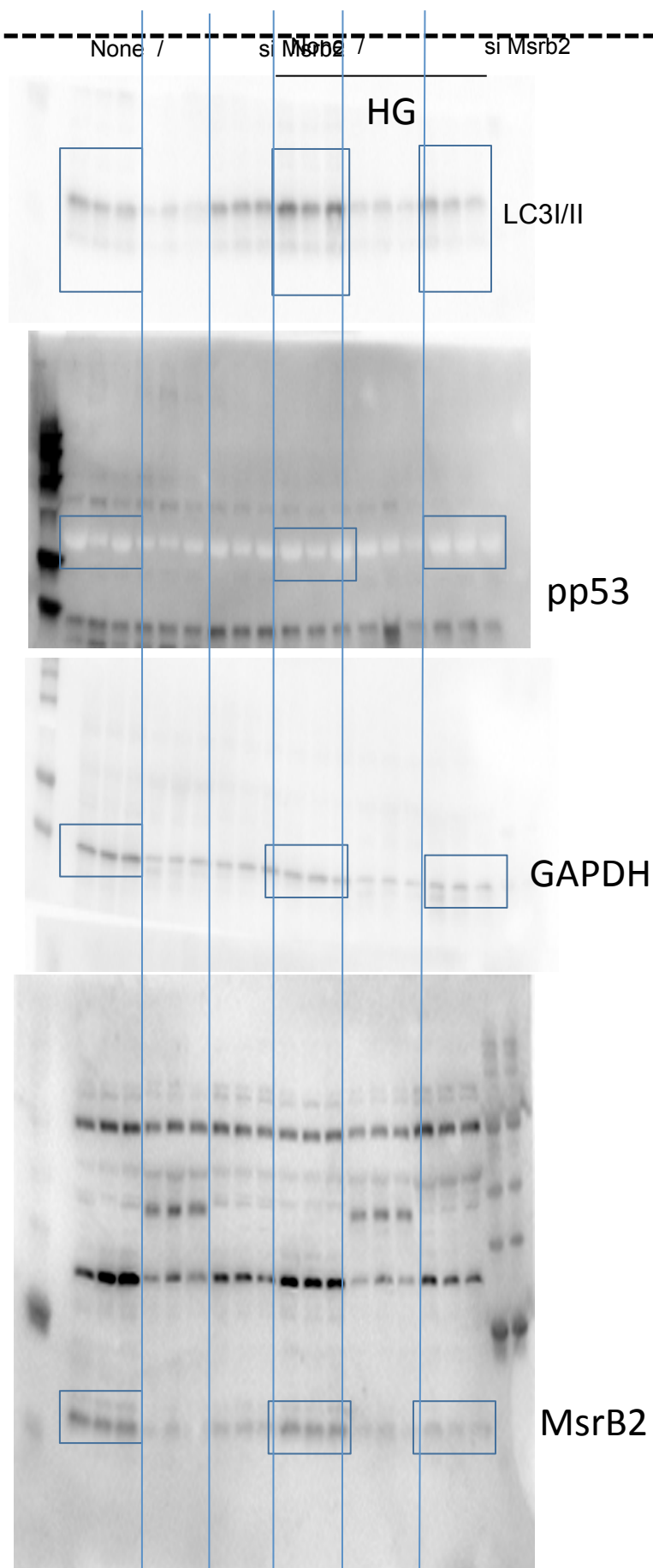

Supplement: Supplementary file 4 — Source Data for Figure 2 [file EMMM-11-e10409-s002.pdf]
