# Supplementary material for: Mitochondrial MsrB2 serves as a switch and transducer for mitophagy
Source: EMBO Mol Med. 2019 Jul 8;11(8):e10409. doi: 10.15252/emmm.201910409 (PMC6685081; doi:10.15252/emmm.201910409)
Supplement: Supplementary file 5 — Source Data for Figure 5 [file EMMM-11-e10409-s003.pdf]

Figure5
B

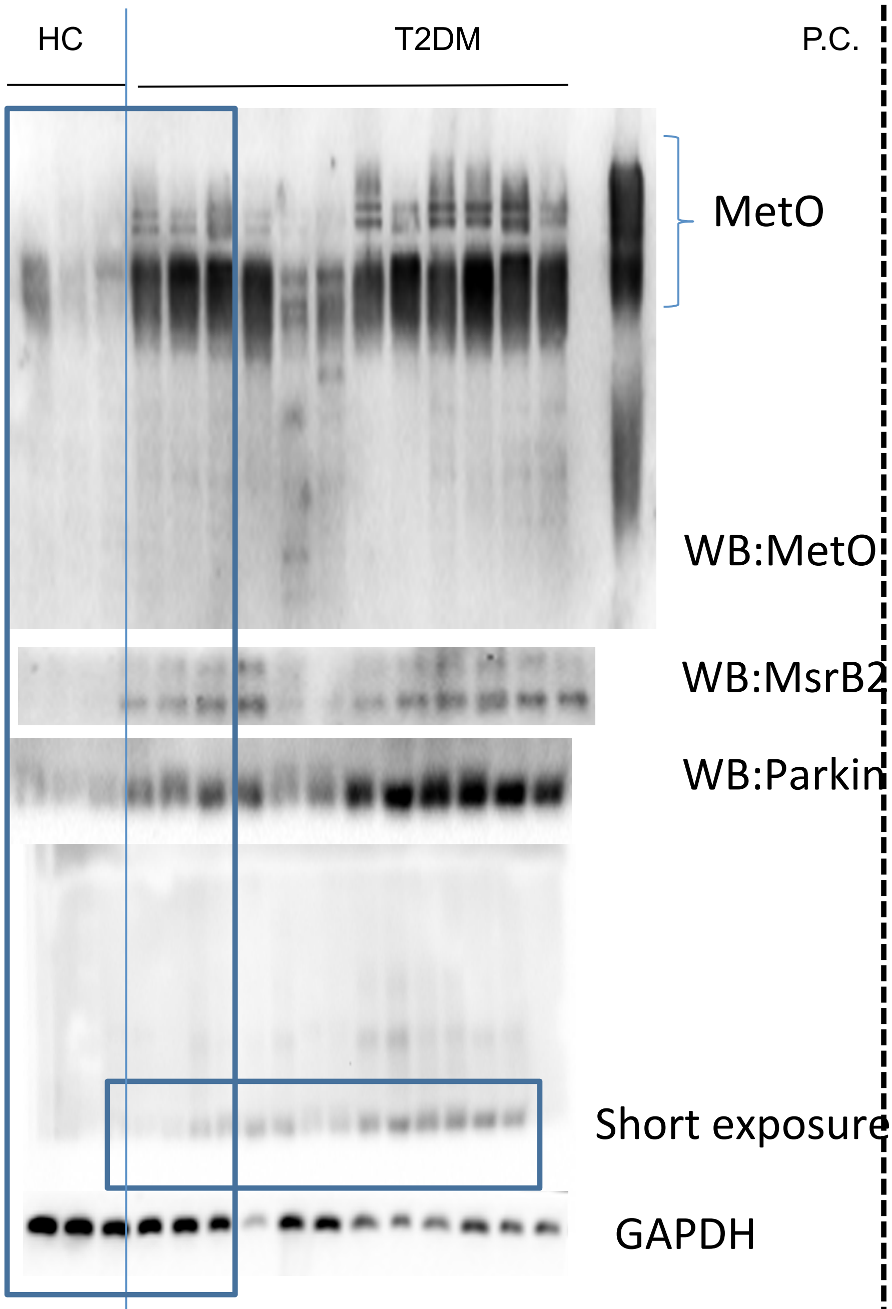
HC
T2DM
P.C.
MetO
WB:MetO
WB:MsrB2
WB:Parkin
Short exposure
GAPDH

Figure5C

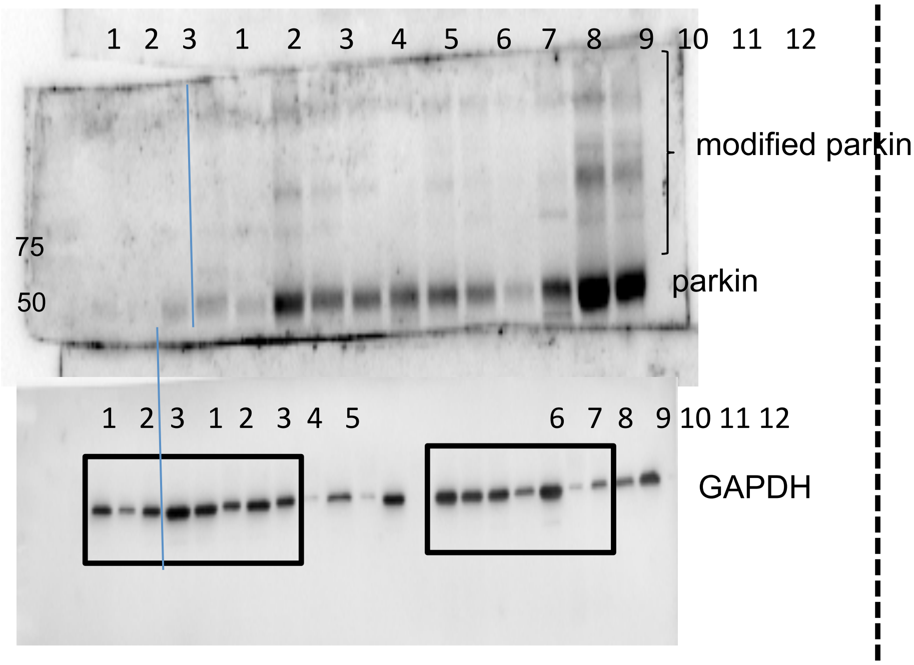
1 2 3 1 2 3 4 5 6 7 8 9 10 11 12
modified parkin
75
50
parkin
1 2 3 1 2 3 4 5 6 7 8 9 10 11 12
GAPDH
